# In silico screening and experimental validation identify riboflavin as an RNA-targeted antiviral against SARS-CoV-2

**DOI:** 10.1038/s41598-025-16949-8

**Published:** 2025-08-22

**Authors:** Chae-Hong Jeong, Yoo Jin Na, Tae Yong Kim, So Young Lee, Jungyeon Kim, Sangmi Ryou

**Affiliations:** Division of Clinical Research, Center for Emerging Virus Research, National Institute of Infectious Diseases, Korea National Institute of Health, 212 Osongsaengmyeong2-ro, Osong-eup, Heungdeok-gu, Cheongju, 28160 Republic of Korea

**Keywords:** Drug repurposing, Antiretroviral, RNA secondary structure, In silico, Riboflavin, SARS-CoV-2, Drug discovery, Small molecules

## Abstract

This study explored drug repurposing strategies against conserved RNA structures in the SARS-CoV-2 genome to address viral mutation challenges. Conserved RNA elements were computationally identified by aligning 283 SARS-CoV-2 genomes from Korean patients. RNA secondary structures were predicted using RNAfold and RNAstructure, followed by virtual screening of 11 compounds using the RNALigands database (binding energy threshold: -6.0 kcal/mol). The antiviral activity and cytotoxicity of riboflavin were experimentally validated in vitro using Vero E6 cells infected with SARS-CoV-2 (MOI 0.01). Riboflavin exhibited selective antiviral activity against SARS-CoV-2 (IC_50_ = 59.41 µM), showing no cytotoxicity at concentrations < 100 µM. Riboflavin treatment during viral inoculation significantly reduced viral replication, whereas riboflavin treatment pre- or post-inoculation had no effect. The other screened compounds lacked antiviral efficacy. In terms of antiviral activity, riboflavin was less potent than remdesivir (IC_50_ = 25.81 µM). Riboflavin is a potential RNA-targeted therapeutic agent against SARS-CoV-2. This study established a framework for integrating computational and experimental methods to identify conserved RNA targets, thus offering a strategy applicable to other RNA viruses. This result indicates the potential of riboflavin as a broad-spectrum antiviral agent against SARS-CoV-2 and highlights the importance of considering nutritional factors in the context of viral infections.

**Supplementary Information:**

The online version contains supplementary material available at 10.1038/s41598-025-16949-8.

## Introduction

The coronavirus disease (COVID-19) pandemic, which emerged in December 2019, posed unprecedented challenges to global health systems and drug development. The lack of effective antiviral drugs in the early stages of the pandemic necessitated the rapid exploration of drug repurposing strategies. These strategies focus on repurposing molecules with known safety profiles to accelerate their development. High-throughput screening, particularly in silico approaches, plays a crucial role in identifying promising candidates for further investigation. This method enables researchers to evaluate the potential of existing drugs for new therapeutic applications efficiently. It has streamlined biological screening processes and enhanced the efficiency of biochemical assays, thereby accelerating the search for effective treatments during the pandemic^1–3^.

In response to the urgent need for effective COVID-19 treatment, various therapeutic molecules, especially immunotherapeutic molecules such as tocilizumab, mavrilimumab, and baricitinib have been explored^4–8^. However, the development of effective drugs requires a comprehensive understanding of virus–drug interactions^9^. A critical challenge in antiviral drug development is overcoming the risk of drug resistance, especially given the high mutation rates of RNA viruses, such as SARS-CoV-2. Consequently, various combination therapies that target multiple stages of the viral life cycle have been explored^10^. Moreover, multitarget strategies that combine RNA- and protein-targeting therapies have emerged as promising approaches for enhancing therapeutic efficacy while minimizing the likelihood of resistance^11^.

Given that SARS-CoV-2 is an RNA virus, its RNA genome serves as the central regulator of viral function. Key structural elements, including the 5′ untranslated region (UTR), frameshift stimulatory element (FSE), and 3′ UTR, play pivotal roles in viral replication and stability, making them prime targets for therapeutic intervention. During the COVID-19 pandemic, computational drug repurposing played a vital role in the efficient identification of therapeutic candidates. Many studies have focused on targeting the key viral proteins involved in SARS-CoV-2 entry and replication^1,12–14^. Other studies have recently highlighted the importance of host-targeted therapies, revealing over 300 host proteins involved in the viral life cycle and underscoring the potential of host-based targets in antiviral drug discovery^15–18^.

This study describes a systematic application of established RNA-targeted drug discovery methodologies to Korean COVID-19 genomic data^19^representing a departure from conventional protein-based therapeutic approaches. Unlike traditional therapies that target viral proteins such as the spike protein (vaccines/antibodies)^20^, main protease (nirmatrelvir)^21^or RNA-dependent RNA polymerase (remdesivir)^22^our approach directly targets conserved functional RNA structures within the viral genome itself. We analyzed 283 SARS-CoV-2 genome sequences obtained from Korean patients to identify conserved RNA structural elements relevant to locally circulating viral strains. This region specific, RNA genome targeting approach provides valuable insights into the genomic characteristics of Korean COVID-19 strains and offers a replicable framework for regional therapeutic strategies^23,24^.

To enhance the specificity and relevance of our RNA-targeted approach, we analyzed conserved structural elements within the SARS-CoV-2 genome isolated from Korean patients. This analysis provides valuable insights into the genomic characteristics of locally circulating viral strains, allowing us to refine our targeted therapeutic strategies.

Considering that the diversity and functionality of RNA within the viral genome is central to RNA-based therapies^25^we screened 11 compounds against the SARS-CoV-2 RNA genome and identified riboflavin (Vitamin B2) as a potentially effective agent. Riboflavin is essential for the synthesis of two major coenzymes involved in energy metabolism, cellular respiration, and antibody production: flavin mononucleotide (FMN) and flavin adenine dinucleotide (FAD). These metabolic functions support the development and activation of the immune cells involved in antiviral defense mechanisms^26^. Our study demonstrated the antiviral activity of riboflavin against SARS-CoV-2 without cytotoxic effects, possibly mediated through the translation or binding processes between host ribosomes and viral RNA. Thus, it contributes to the growing body of evidence supporting RNA-targeted therapies as promising avenues for antiviral drug development^14,27^.

## Results

### Identification and analysis of conserved RNA structures in SARS-CoV-2 genome and screening of potential RNA-binding small molecules

Using sequence alignments of 283 SARS-CoV-2 sequences, we ranked the genomic regions by RNA sequence conservation, identifying 10 conserved regions of at least 15 nucleotides that exactly matched the SARS-CoV-2 reference sequence. Additionally, we analyzed the secondary structure by incorporating sequences from the conserved SARS-CoV-2 sequence to explore evolutionary conservation^28^. To predict the secondary structures of these conserved regions, we used two computational tools, RNAfold^29^ and RNAstructure^30^with their default parameters. We compared the predicted structures using both tools to assess consistency and identify any significant differences. The secondary structures of the ten conserved sequences were predicted and visualized using these tools. We used the RNALigands databse^25,31^ to screen for small molecules that could potentially interact with the predicted RNA secondary structures by comparing the ligand structure, chemical properties, RNA sequence, and RNA secondary structure with those of existing ligands or RNA. To narrow down the candidates, we selected ligands with high binding scores based on their interactions with RNA sequences from the PDB. Ligands that appeared repeatedly across multiple screening analyses were also included. Identification of these top ligands represents a significant step forward in the development of RNA-targeting therapeutics. Finally, we identified 11 chemicals that bind to RNA secondary structure motifs (Table 1 and Supplementary Table S1).

**Table 1:** Top 10 RNA secondary motifs and chemicals predicted by the RNALigands database.

Name	Sequence, secondary structure	Binding sites	Motif	PDB (score)	Chemical
1	AUUAAAGGUUUAUACCUUCCCAGGUAACAAACCAACCAACUUUCGAUCUCUUGUAGAUCUGUUCUCUAAACGAACUUUAAAAUCUGUGUGGCUGUCACUCGGCUG ……(((((.(((((….))))).)))))……….(((((….))))).((((…….))))………….(((((….))))). (− 19.50)	5′UTR	I: (U, A) A (U, A) AC, H: (G, C) UCACU	**FMN(106)**, B1Z(49)	**Riboflavin 5'-monophosphate sodium salt hydrate**
2	AUGCUUAGUGCACUCACGCAGUAUAAUUAAUAACUAAUUACUGUCGUUGACAGGACACGAGUAACUCGUCUAUCUUCUGCAGGCUGCUUACGGUUUCGUCCGUGUUGCAGCCGAUCAUCAGCACAUCUAGGUUU .(((((((((….((((((((……………))))).)))((.(((((.(((((…)))))….)))))))((((((.(((((……))))).))))))…….))))…))))). (− 38.30)	5′UTR	B: (G, C) U (C, G), B: (G, C) A (C, G), H: (G, C) UAA, I: (U, A) CUAUC (U, A) C, I: (C, G) U (U, G) UU, H: (G, C) UUUCGU, B: (C, G) AUC (U, A), M: (C, G) ACUC (A, U) (U, A) (G, C) GAUCAUCA	GLY(86), **TOA(92)**,** 4PQ(23)**,** SAH(39)**, SAM(32), **HPA(99)**, C2E(38), SAM(54)	3-Ammonio-3-deoxy-alpha-D-glucopyranose S-Adenosyl-l-homocysteine **Hypoxanthine** 5-Hydroxy-l-tryptophan
3	GUCCGGGUGUGACCGAAAGGUAA …(((……)))……. (− 4.30)	5′UTR	H: (G, C) GUGUGA	ARG(25)	
4	CGGUGUAAGUGCAGCCCGUCUUACACCGUGCGGCACAGGCACUAGUACUGAUGUCGUAUACAGGGC .((((((((.((….))))))))))(((((((((((………))).))))))))……. (− 24.00)	FSE	B: (G, C) U (G, U), B: (G, C) A (U, A)	LYS(48), **TOA(58)**	Kanosamine hydrochloride
5	UGUGCAGAAUGAAUU …………… (0.00)	FSE	–	–	
6	UCGUAACUACAUAGCACAAGUA …………………. (0.00)	FSE	–	–	
7	CAAUCUUUAAUCAGUGUGUAA ………………… (0.00)	3′UTR	–	–	
8	AUUAGGGAGGACUUGAAAGAGCCACCACAUUUUCACCGA ….((.((.(((….))))).))…………. (− 5.60)	3′UTR	I: (G, C) GA (G, C) A, B: (G, C) A (C, G), H: (U, G) GAAA	GES(60), **TOA(92)**, AMZ(60)	3-Ammonio-3-deoxy-alpha-D-glucopyranose
9	AGUGUACAGUGAACAAUGCUAGG …………………. (0.00)	3′UTR	–	–	
10	AGAGCUGCCUAUAUGGAAGAGCCCUAAUGUGUAAAAUUAAUUUUAGUAGUGCUAUCCCCAUGUGAUUUUAAUAGCUUCUUAGGAGAAUGACAAAAAAAAAAAAAAAAAAAAAAAAAAAAAAAA .((((((.(((((((…(((.(((.((………….)).))).)))….)))))))…….))))))………………………………………. (− 12.10)	3′UTR	I: (C, G) C (C, G) U, I: (A, U) A (U, A) G	GES(71**)**,** HPA(89)**	**Hypoxanthine**

FMN, FLAVIN MONONUCLEOTIDE; B1Z, adenosylcobalamin; GLY, GLYCINE; TOA, 3-ammonio-3-deoxy-alpha-D-glucopyranose; 4PQ, 5-hydroxy-L-tryptophan; SAH, S-ADENOSYL HOMOCYSTEINE; SAM, S-ADENOSYL METHIONINE; HPA, HYPOXANTHINE; C2E, 9,9′-[(2R,3R,3aS,5 S,7aR,9R,10R,10aS,12 S,14aR)-3,5,10,12-tetrahydroxy-5,12-dioxidooctahydro-2 H,7 H-difuro[3,2-d:3′,2′j][1,3,7,9,2,8]tetraoxadiphosphacyclododecine-2,9-diyl]bis(2-amino-1,9-dihydro-6 H-purin-6-one); ARG, ARGININE; LYS, LYSINE; GES, 3-deoxy-4-C-methyl-3-(methylamino)-beta-L-arabinopyranose; AMZ, AMINOIMIDAZOLE 4-CARBOXAMIDE RIBONUCLEOTIDE. The compounds highlighted in bold represent candidates selected based on our screening criteria.

### Cytotoxicity and antiviral activity screening of computationally predicted drugs against SARS-CoV-2 in Vero E6 cells

**Fig. 1:**
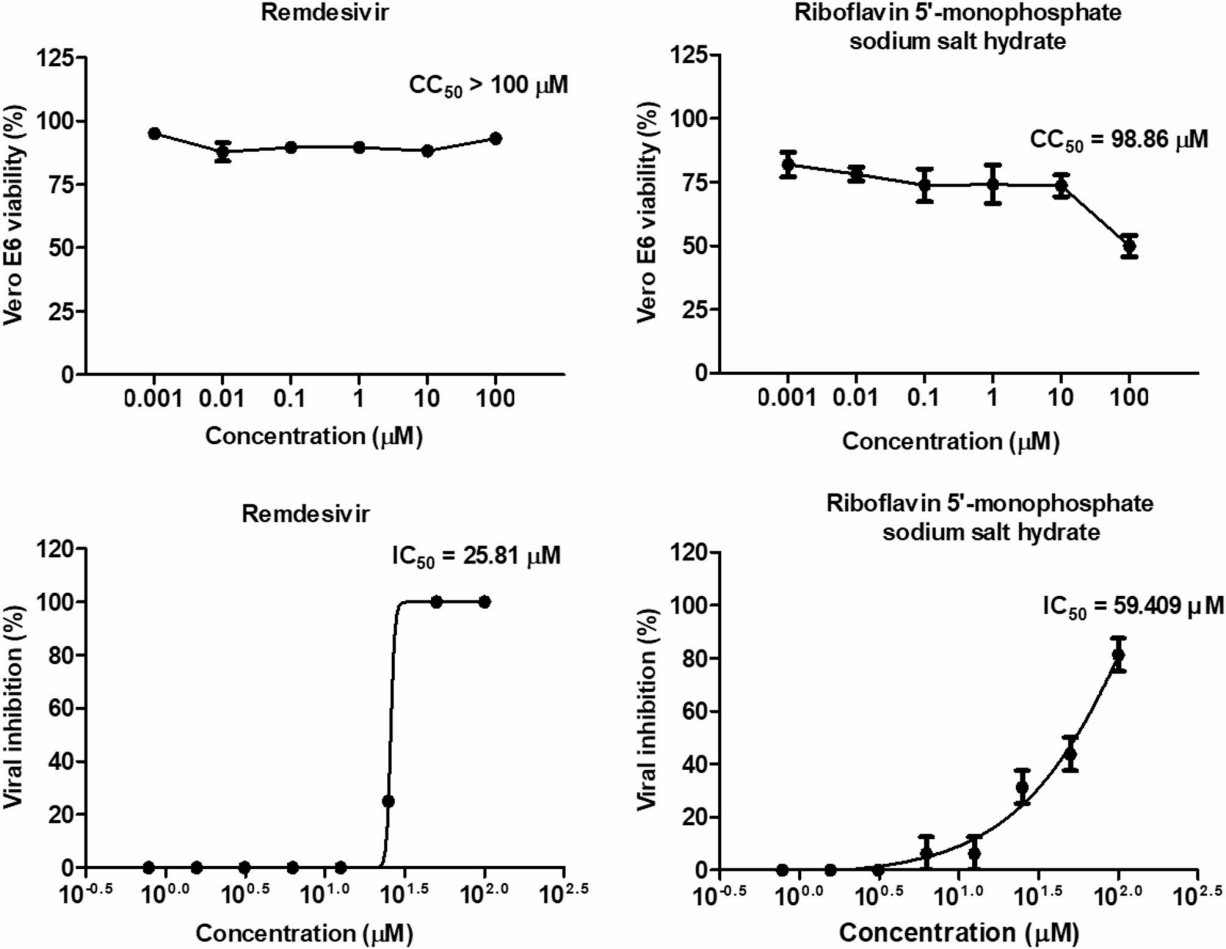
Evaluation of the cytotoxicity and antiviral efficacy of remdesivir and FMN, a riboflavin derivative, in Vero E6 cells. Vero E6 cells were treated with indicated doses of remdesivir and FMN. CC_50_ and IC_50_ are indicated on the curves. Cells were treated with remdesivir or FMN for 2 h and then maintained in drug-free media for 48 h. Cell viability (**a**) and antiviral activity (**b**) were evaluated using an EZ-Cytox kit and plaque assay with crystal violet staining, respectively.

Eleven drugs were tested for cytotoxicity in Vero E6 cells. The drugs were evaluated at concentrations ranging from 1 nM to 100 µM in 10-fold serial dilutions to determine their dose-dependent effects on cell viability. Figure 1 and Supplementary Table S2 show the 50% cytotoxic concentration (CC_50_) of the 11 drugs after 2 days of treatment. Riboflavin and several other drugs exhibited CC_50_ exceeding 100 µM, indicating no observable toxicity in Vero E6 cells within the investigated concentration range. To assess the overall antiviral efficacy of the computationally predicted drugs in vitro, Vero E6 cells were infected with SARS-CoV-2, and the half-maximal inhibitory concentrations (IC_50_) of each drug was determined. Among the compounds tested, riboflavin exhibited inhibitory effects against SARS-CoV-2 at high micromolar concentrations (IC_50_ = 59.41 µM). However, the other ten drugs showed no significant antiviral effects against SARS-CoV-2 at the tested concentrations. Our study demonstrated that riboflavin exhibits selective antiviral activity against SARS-CoV-2 in Vero E6 cells, with riboflavin administration during viral inoculation showing significant inhibition, whereas riboflavin treatment at 2 h prior to infection and 2 h post-infection displayed no measurable antiviral effects (Supplementary Table S3). Furthermore, when riboflavin was added to the culture medium after viral inoculation, additional inhibitory effects on viral replication were observed (Supplementary Fig. S1). Remdesivir was used as a positive control in this assay, and its IC_50_ values were compared.

## Discussion

The primary objective of this study was to explore a structure-based drug repurposing strategy that targets conserved RNA elements in the SARS-CoV-2 genome. Through the computational screening of 11 compounds, we identified riboflavin as a promising antiviral candidate. These findings not only validate the potential of RNA-targeted approaches for antiviral drug development but also emphasize the unique role of nutritional components, such as riboflavin, in addressing viral infections, such as SARS-CoV-2 infection. However, riboflavin exhibited lower direct antiviral effects against SARS-CoV-2 than anticipated, requiring higher concentrations for significant inhibition (IC_50_ = 59.4 µM) compared to remdesivir (IC_50_ = 25.8 µM). While riboflavin’s potency is considerably lower, its lack of cytotoxicity at concentrations ≤ 100 µM (CC_50_ > 100 µM) and minimal hepatorenal toxicity position it as a safer candidate for long-term or prophylactic use^32^. This advantage is particularly relevant given reports of remdesivir-associated hepatotoxicity (e.g., elevated liver enzymes in 35% of all patients) and nephrotoxicity risks in clinical settings^33^.

This is consistent with the results of a recent study by Akasov et al., who reported the predominant anti-inflammatory effects of riboflavin over direct viral suppression in Vero E6 cells. Notably, while riboflavin was found to inhibit SARS-CoV-2 papain-like protease in vitro (> 50 µM), it fails to block viral replication in cellular models, suggesting that its mechanism may target early viral entry stages rather than late assembly processes^32,34^. This observation may be explained by several factors. The binding of riboflavin to the viral genome might not sufficiently impair crucial functions, possibly necessitating higher concentrations for effective inhibition. Additionally, the rapid intracellular metabolism of riboflavin, combined with its limited membrane permeability and photosensitivity, may result in suboptimal intracellular concentrations. Therefore, the antiviral action of riboflavin may be attributed to its anti-inflammatory properties rather than direct antiviral activity.

While computational RNA docking provided the initial rationale for compound selection, our experimental results cannot definitively establish direct RNA binding as the primary mechanism of action. The observed antiviral effects may result from multiple complementary mechanisms, including potential RNA interactions, NF-κB inhibition, inflammasome suppression, and antioxidant actions^35–40^. Our findings suggest that riboflavin’s antiviral activity against SARS-CoV-2 may be mediated through multiple complementary mechanisms, particularly via its immunomodulatory properties, rather than through a singular direct antiviral effect. Literature evidence suggests that riboflavin’s therapeutic potential likely stems from its well-established immunomodulatory properties, including NF-κB pathway inhibition, inflammasome regulation^36,39^and antioxidant enzyme support through FAD/FMN cofactor provision^38^. These mechanisms directly address COVID-19’s core pathophysiology including cytokine storm and immune dysregulation^41,42^providing a stronger scientific rationale for riboflavin’s therapeutic potential than RNA binding alone^34^. Moreover, Clinical evidence supports these immunomodulatory mechanisms, as riboflavin supplementation has been associated with improved immune markers in COVID-19 patients^35^suggesting practical therapeutic applicability beyond the RNA-targeting hypothesis (Supplementary Fig. S2).

Cardoso et al. found that riboflavin competitively inhibited viral proteases, with IC_50_ values in the medium micromolar range for ZIKV and YFV proteases, and potent activity against CHIKV nsP2 protease^43,44^. Although these findings are not directly related to SARS-CoV-2, they suggest that the antiviral potential of riboflavin varies depending on the specific virus and target protein, further highlighting the complexity of its mechanism of action.

Understanding the precise molecular mechanisms underlying the antiviral effects of riboflavin may lead to targeted modifications that enhance its efficacy. Despite these challenges, the lack of cytotoxicity observed with riboflavin, even at high concentrations, warrants further investigation into its potential as an antiviral agent. However, further studies are required to determine the actual therapeutic index and efficacy at clinically relevant doses.

The strength of our study lies in the fact that, by focusing on conserved RNA structures, we addressed the challenge of viral mutations and offered a potentially more sustainable solution for the long-term management of viral infections, not only for SARS-CoV-2 but also for other RNA viruses. Furthermore, this study demonstrated the power of integrating computational approaches with experimental validation to accelerate drug discovery^28,29^. By leveraging in silico screening methods and structural biology insights, we rapidly identified promising candidates for further investigation, providing an efficient model for drug repurposing in the face of emerging infectious diseases. This integrated approach, from population-specific genomic analysis through computational screening to experimental validation, provides are replicable framework for identifying repurposed therapeutics.

Several important limitations must be acknowledged. First, while computational modeling predicted riboflavin-RNA interactions, we lack direct experimental validation of these binding events in cellular systems. Second, the antiviral effects observed may result from multiple cellular mechanisms operating independently or synergistically, rather than solely through RNA targeting. Third, the photosensitivity and cellular uptake characteristics of riboflavin prevented reliable assessment of direct RNA-targeting activity in our experimental system. Future investigations should include biochemical binding assays, structure-activity relationship studies, and comprehensive pathway analyses to determine the primary mechanism(s) responsible for riboflavin’s antiviral properties. In conclusion, this study provides a potential therapeutic approach for COVID-19 and establishes a framework for identifying antiviral agents targeting conserved RNA structures in viral genomes. This approach could potentially be applied to other RNA viruses, leading to the development of broad-spectrum antiviral therapies. Further research is necessary to elucidate these mechanisms and optimize the potential of riboflavin as a therapeutic agent. As we continue to face the challenges posed by SARS-CoV-2 and future viral threats, the exploration of novel strategies such as RNA-targeted therapies and the consideration of nutritional factors in antiviral responses opens new avenues for research and therapeutic development in infectious disease management.

## Methods

### Genomic analysis and identification of the conserved RNA structures in SARS-CoV-2

The 283 SARS-CoV-2 genome sequences and resources used in this study were obtained from the Establishment of Multi-Omics Data Related with COVID-19 (CODA-D23017), distributed by the National Biobank of Korea (NBK-2022-066) under the Agency for Disease Control and Prevention. Raw sequencing data were initially processed using FastQC (v0.11.9) to assess the read quality. Trimmomatic (v0.39) was used to remove low-quality reads and adapter sequences. The cleaned reads were aligned to the human reference genome (GRCh38) using HISAT2 (v2.2.1) to remove host sequences. Unmapped reads were aligned to the SARS-CoV-2 reference genome (GenBank accession number: NC_045512.2) using HISAT2 with the default settings. The resulting SAM files were converted to BAM format, sorted, and indexed using SAMtools (v1.11). The average depth of coverage across all samples was 1000X, with a minimum coverage of 100X required for inclusion in further analysis. Variant calling was performed using SAMtools, and consensus sequences for each sample were generated using BCFtools consensus with a base quality threshold of 20 and read depth of at least 10. Seqtk (v1.3) was used to convert the resulting FASTQ files to FASTA format. Multiple sequence alignment of the 283 consensus sequences was performed using Clustal Omega (v1.2.4) with the default parameters. Gaps in the alignment were treated as non-conserved positions. The resulting alignment was analyzed using a custom Python script to identify conserved regions. Conserved regions were defined as contiguous stretches of 15 or more nucleotides that were at least 98% conserved across all sequences, allowing up to 2% gaps or mismatches. The statistical significance of the conserved regions was assessed using a permutation test with 1000 randomizations of the sequence alignments. For each permutation, the nucleotide positions within each sequence were randomly shuffled, and the number of conserved regions was calculated. The P-value for each observed conserved region was calculated as the proportion of permutations that produced an equal or greater number of conserved regions of the same or greater length.

### Secondary structure of RNA bound to small-molecule ligands

Ten of the longest conserved regions were selected for predicting RNA secondary structure. Two tools were used to ensure the robustness of the predictions. First, RNAfold (ViennaRNA Package 2.4.14) was used to predict the structures using the Minimum Free Energy (MFE) algorithm with Turner 2004 energy parameters. Next, RNAstructure (v6.2) was used with three different algorithms (MFE, MaxExpect, and ProbKnot). The structures predicted using these methods were compared and analyzed for each conserved region. A consensus structure was derived when at least two methods predicted similar structural elements. The RNALigands database (version 2.0) was used to screen for potential small-molecule interactions, with a binding energy threshold of -6.0 kcal/mol. Following the initial RNALigands screening, compounds were selected for experimental validation based on multiple criteria. The RNALigands program employs a scoring system based on algorithms for RNA secondary structure motif alignment, inspired by the Needleman Wunsch algorithm and considering both RNA sequence identity and secondary structure information. The score is calculated by integrating the SNW (Needleman Wunsch alignment score), nucleotide substitution scores, base pair substitution scores, and loop cost deviations. Higher scores indicate more similar RNA motif structures, suggesting higher probability of binding with similar ligands. This screening method demonstrated 77% accuracy in benchmark testing^25^. Based on the RNALigands scoring results, we selected ligands that were commonly identified in both PDB and R-BIND databases and exhibited high scores.

### Cells and viruses

Vero E6 cells (ATCC CRL-1587) were purchased from the American Type Culture Collection (ATCC).

They were cultured in Dulbecco’s modified Eagle medium (Gibco) supplemented with 10% fetal bovine serum and 1% penicillin-streptomycin and maintained in a humidified incubator at 37 °C with 5% CO_2_. SARS-CoV-2 (S-clade strain NCCP43331) was provided by the Korea Centers for Disease Control and Prevention. Viral infection and assays were performed in a biosafety level 3 facility. Cells were used within 20 passages and regularly tested for mycoplasma contamination. Virus stocks were prepared by infecting Vero E6 cells at a multiplicity of infection (MOI) of 0.01 and harvesting the supernatant after 72 h. Virus titers were determined using plaque assay and expressed as plaque-forming units per mL.

### Compounds

c-di-AMP sodium salt (cat. SML1231), 5-hydroxy-L-tryptophan (cat. PHL80477), adenine (cat. A2786), cGAMP sodium salt (cat. SML1232), hypoxanthine (cat. H9377), 6,7-dimethoxy-2-(1-piperazinyl)-4-quinazolinamine (cat. R733466), coenzyme B12 (cat. C0884), riboflavin 5′-monophosphate sodium salt hydrate (cat. F8399), neomycin solution (cat. N1142), and hydroxocobalamin (cat. H1428000) were purchased from Sigma-Aldrich. S-adenosyl-l-homocysteine (cat. 1012112) was purchased from USP. Remdesivir (cat. HY-104077) was purchased from MedChem Express. These compounds were dissolved in dimethyl sulfoxide (DMSO; Sigma-Aldrich) or deionized water to achieve a final concentration of 10–100 mM. All compounds were stored according to the manufacturer’s recommendations. DMSO-dissolved compounds were aliquoted and stored at -20 °C, while water-dissolved compounds were stored at 4 °C.

### Determination of cell viability and 50% cytotoxic concentration

To determine the cytotoxicity of the compounds, cell viability was measured using an EZ-Cytox kit (Daeil Lab Service) according to the manufacturer’s instructions. Vero E6 cells were seeded into 96-well plates at 5 × 10^3^ cells/well. The cells were cultured for 1 day, followed by treatment with 10-fold serial dilutions of compounds (1 nM–100 µM) and incubation at 37 °C for 2 days. EZ-Cytox solution was added to each well and their contents were incubated for 2 h. Subsequently, the absorbance of each well was measured at 450 nm using a microplate reader (SpectraMax i3x, Molecular Devices) with a reference wavelength of 650 nm. The CC_50_ was calculated using the GraphPad Prism 5 software (GraphPad). Each experiment was performed in triplicates and repeated independently. CC_50_ was calculated using a nonlinear regression model.

### Determination of half-maximal inhibitory concentration

Infection assays were performed to determine the IC_50_ of the compounds. Vero E6 cells were seeded into 96-well plates (1.5 × 10^4^ cells/well) and incubated for 18 h. The cells were simultaneously treated with 2-fold serial dilutions of the compounds (0.1 nM–100 µM) and infected with SARS-CoV-2 at an MOI of 0.01 for 2 h. After infection, the cells were washed three times with phosphate-buffered saline and incubated in fresh medium for 48 h. Viral titers in the supernatants were determined by performing a plaque assay using crystal violet staining. The percentage of viral inhibition was calculated relative to DMSO-treated controls. The IC_50_ values were determined by nonlinear regression analysis using GraphPad Prism 8.0. Each experiment was performed in triplicate and independently repeated twice.

## Supplementary Information

Below is the link to the electronic supplementary material.

Supplementary Material 1

## Data Availability

All data generated during this study are included in this published article and its supplementary information files.

## References

[1] Chakraborty, C., Sharma, A. R., Bhattacharya, M., Agoramoorthy, G. & Lee, S. S. The drug repurposing for COVID-19 clinical trials provide very effective therapeutic combinations: lessons learned from major clinical studies. *Front. Pharmacol.* **12**, 704205 (2021). 34867318 10.3389/fphar.2021.704205 PMC8636940

[2] Pushpakom, S. et al. Drug repurposing: progress, challenges and recommendations. *Nat. Rev. Drug Discovery*. **18**, 41–58 (2019). 30310233 10.1038/nrd.2018.168

[3] Rameshrad, M., Ghafoori, M., Mohammadpour, A. H., Nayeri, M. J. D. & Hosseinzadeh, H. A comprehensive review on drug repositioning against coronavirus disease 2019 (COVID19). *Naunyn Schmiedebergs Arch. Pharmacol.* **393**, 1137–1152. 10.1007/s00210-020-01901-6 (2020). 32430617 10.1007/s00210-020-01901-6 PMC7235439

[4] Lucaj, T. et al. An overview of the development of pharmacotherapeutics targeting SARS-CoV-2. *Drug Discovery Today*, 104126 (2024). 10.1016/j.drudis.2024.104126 39097220

[5] Del Valle, D. M. et al. An inflammatory cytokine signature predicts COVID-19 severity and survival. *Nat. Med.* **26**, 1636–1643. 10.1038/s41591-020-1051-9 (2020). 32839624 10.1038/s41591-020-1051-9 PMC7869028

[6] Iserman, C. et al. Genomic RNA Elements Drive Phase Separation of the SARS-CoV-2 Nucleocapsid. *Mol Cell* 80, 1078–1091 e1076, (2020). 10.1016/j.molcel.2020.11.041 10.1016/j.molcel.2020.11.041 PMC7691212 33290746

[7] Hashemian, S. M. R. et al. Paxlovid (Nirmatrelvir/Ritonavir): A new approach to Covid-19 therapy? *Biomed. Pharmacother.* **162**, 114367 (2023). 37018987 10.1016/j.biopha.2023.114367 PMC9899776

[8] Harris, M. & Bagozzi, D. WHO discontinues hydroxychloroquine and lopinavir/ritonavir treatment arms for COVID-19. *World Health Organ. News Release* **4** (2020).

[9] Yousefi, H., Mashouri, L., Okpechi, S. C., Alahari, N. & Alahari, S. K. Repurposing existing drugs for the treatment of COVID-19/SARS-CoV-2 infection: A review describing drug mechanisms of action. *Biochem. Pharmacol.* **183**, 114296 (2021). 33191206 10.1016/j.bcp.2020.114296 PMC7581400

[10] Hijikata, A. et al. Current status of structure-based drug repurposing against COVID-19 by targeting SARS-CoV-2 proteins. *Biophys. Physicobiology*. **18**, 226–240 (2021). 10.2142/biophysico.bppb-v18.025 PMC8550875 34745807

[11] Rodrigues, L., Cunha, B., Vassilevskaia, R., Viveiros, T., Cunha, C. & M. & Drug repurposing for COVID-19: A review and a novel strategy to identify new targets and potential drug candidates. *Molecules* **27**10.3390/molecules27092723 (2022). 10.3390/molecules27092723 PMC9099573 35566073

[12] Galindez, G. et al. Lessons from the COVID-19 pandemic for advancing computational drug repurposing strategies. *Nat. Comput. Sci.* **1**, 33–41 (2021). 38217166 10.1038/s43588-020-00007-6

[13] Owen, D. R. et al. An oral SARS-CoV-2 M(pro) inhibitor clinical candidate for the treatment of COVID-19. *Science* **374**, 1586–1593. 10.1126/science.abl4784 (2021). 34726479 10.1126/science.abl4784

[14] Hegde, S., Tang, Z., Zhao, J. & Wang, J. Inhibition of SARS-CoV-2 by targeting conserved viral RNA structures and sequences. *Front. Chem.* **9**, 802766. 10.3389/fchem.2021.802766 (2021). 35004621 10.3389/fchem.2021.802766 PMC8733332

[15] Li, S., Li, H., Lian, R., Xie, J. & Feng, R. New perspective of small-molecule antiviral drugs development for RNA viruses. *Virology*, 110042 (2024). 10.1016/j.virol.2024.110042 38492519

[16] Lei, S., Chen, X., Wu, J., Duan, X. & Men, K. Small molecules in the treatment of COVID-19. *Signal. Transduct. Target. Therapy*. **7**, 387 (2022). 10.1038/s41392-022-01249-8 PMC9719906 36464706

[17] Gordon, D. E. et al. Comparative host-coronavirus protein interaction networks reveal pan-viral disease mechanisms. *Science* **370**, eabe9403 (2020). 33060197 10.1126/science.abe9403 PMC7808408

[18] Nalewaj, M. & Szabat, M. Examples of structural motifs in viral genomes and approaches for RNA structure characterization. *Int. J. Mol. Sci.* **23**, 15917 (2022). 36555559 10.3390/ijms232415917 PMC9784701

[19] Jo, H. Y. et al. Establishment of the large-scale longitudinal multi-omics dataset in COVID-19 patients: data profile and biospecimen. *BMB Rep.* **55**, 465–471. 10.5483/BMBRep.2022.55.9.077 (2022). 35996834 10.5483/BMBRep.2022.55.9.077 PMC9537027

[20] Polack, F. P. et al. Safety and efficacy of the BNT162b2 mRNA Covid-19 vaccine. *N Engl. J. Med.* **383**, 2603–2615. 10.1056/NEJMoa2034577 (2020). 33301246 10.1056/NEJMoa2034577 PMC7745181

[21] Hammond, J. et al. Oral nirmatrelvir for High-Risk, nonhospitalized adults with Covid-19. *N Engl. J. Med.* **386**, 1397–1408. 10.1056/NEJMoa2118542 (2022). 35172054 10.1056/NEJMoa2118542 PMC8908851

[22] Beigel, J. H. et al. Remdesivir for the treatment of Covid-19 - Final report. *N Engl. J. Med.* **383**, 1813–1826. 10.1056/NEJMoa2007764 (2020). 32445440 10.1056/NEJMoa2007764 PMC7262788

[23] Cousins, H. C., Nayar, G. & Altman, R. B. Computational approaches to drug repurposing: methods, challenges, and opportunities. *Annu. Rev. Biomed. Data Sci.* **7**, 15–29. 10.1146/annurev-biodatasci-110123-025333 (2024). 38598857 10.1146/annurev-biodatasci-110123-025333

[24] Nguyen, T. K. et al. The therapeutic landscape for COVID-19 and post-COVID-19 medications from genetic profiling of the Vietnamese population and a predictive model of drug-drug interaction for comorbid COVID-19 patients. *Heliyon* **10**, e27043. 10.1016/j.heliyon.2024.e27043 (2024). 38509882 10.1016/j.heliyon.2024.e27043 PMC10950508

[25] Sun, S., Yang, J. & Zhang, Z. RNALigands: a database and web server for RNA–ligand interactions. *Rna* **28**, 115–122 (2022). 34732566 10.1261/rna.078889.121 PMC8906548

[26] Maggini, S., Wintergerst, E. S., Beveridge, S. & Hornig, D. H. Selected vitamins and trace elements support immune function by strengthening epithelial barriers and cellular and humoral immune responses. *Br. J. Nutr.* **98**, S29–S35 (2007). 17922955 10.1017/S0007114507832971

[27] Zhu, S., Rooney, S. & Michlewski, G. RNA-targeted therapies and high-throughput screening methods. *Int. J. Mol. Sci.* **21**, 2996 (2020). 32340368 10.3390/ijms21082996 PMC7216119

[28] Sharma, P. P. et al. Computational methods directed towards drug repurposing for COVID-19: advantages and limitations. *RSC Adv.* **11**, 36181–36198 (2021). 35492747 10.1039/d1ra05320e PMC9043418

[29] Cetin, Y. et al. Review on in Silico methods, high-throughput screening techniques, and cell culture based in vitro assays for SARS-CoV-2. *Curr. Med. Chem.* **29**, 5925–5948 (2022). 35761502 10.2174/0929867329666220627121416

[30] Rangan, R., Zheludev, I. N. & Das, R. RNA genome conservation and secondary structure in SARS-CoV-2 and SARS-related viruses. *BioRxiv*10.1101/2020.03.27.012906 (2020). 32398273 10.1261/rna.076141.120 PMC7373990

[31] Philips, A., Milanowska, K., Lach, G. & Bujnicki, J. M. LigandRNA: computational predictor of RNA-ligand interactions. *RNA* **19**, 1605–1616. 10.1261/rna.039834.113 (2013). 24145824 10.1261/rna.039834.113 PMC3860260

[32] Lorenz, R. et al. ViennaRNA package 2.0. *Algorithms Mol. Biol.* **6**10.1186/1748-7188-6-26 (2011). 10.1186/1748-7188-6-26 PMC3319429 22115189

[33] Reuter, J. S. & Mathews, D. H. RNAstructure: software for RNA secondary structure prediction and analysis. *BMC Bioinform.* **11**, 1–9 (2010). 10.1186/1471-2105-11-129 PMC2984261 20230624

[34] Akasov, R. A. et al. Evaluation of molecular mechanisms of riboflavin anti-COVID-19 action reveals anti-inflammatory efficacy rather than antiviral activity. *Biochim. Biophys. Acta Gen. Subj.* **1868**, 130582. 10.1016/j.bbagen.2024.130582 (2024). 38340879 10.1016/j.bbagen.2024.130582

[35] Akasov, R. A. et al. Riboflavin for COVID-19 adjuvant treatment in patients with mental health disorders: observational study. *Front. Pharmacol.* **13**, 755745. 10.3389/fphar.2022.755745 (2022). 35359854 10.3389/fphar.2022.755745 PMC8960625

[36] Suwannasom, N., Kao, I., Pruss, A., Georgieva, R. & Baumler, H. Riboflavin: the health benefits of a forgotten natural vitamin. *Int. J. Mol. Sci.* **21**10.3390/ijms21030950 (2020). 10.3390/ijms21030950 PMC7037471 32023913

[37] Lauhon, C. T. & Szostak, J. W. RNA aptamers that bind flavin and nicotinamide redox cofactors. *J. Am. Chem. Soc.* **117**, 1246–1257. 10.1021/ja00109a008 (1995). 11539282 10.1021/ja00109a008

[38] Dricot, C. et al. Riboflavin for women’s health and emerging microbiome strategies. *NPJ Biofilms Microbiomes*. **10**10.1038/s41522-024-00579-5 (2024). 10.1038/s41522-024-00579-5 PMC11486906 39420006

[39] Ahn, H., Lee, G. S. & Riboflavin Vitamin B2, attenuates NLRP3, NLRC4, AIM2, and non-canonical inflammasomes by the Inhibition of caspase-1 activity. *Sci. Rep.* **10**, 19091. 10.1038/s41598-020-76251-7 (2020). 33154451 10.1038/s41598-020-76251-7 PMC7645791

[40] Qureshi, A. A. et al. Suppression of nitric oxide induction and pro-inflammatory cytokines by novel proteasome inhibitors in various experimental models. *Lipids Health Dis.* **10**, 177. 10.1186/1476-511X-10-177 (2011). 21992595 10.1186/1476-511X-10-177 PMC3206449

[41] Tay, M. Z., Poh, C. M., Renia, L., MacAry, P. A. & Ng, L. F. P. The trinity of COVID-19: immunity, inflammation and intervention. *Nat. Rev. Immunol.* **20**, 363–374. 10.1038/s41577-020-0311-8 (2020). 32346093 10.1038/s41577-020-0311-8 PMC7187672

[42] Hu, B., Huang, S. & Yin, L. The cytokine storm and COVID-19. *J. Med. Virol.* **93**, 250–256. 10.1002/jmv.26232 (2021). 32592501 10.1002/jmv.26232 PMC7361342

[43] Reuter, J. S. & Mathews, D. H. RNAstructure: software for RNA secondary structure prediction and analysis. *BMC Bioinform.* **11**, 129. 10.1186/1471-2105-11-129 (2010). 10.1186/1471-2105-11-129 PMC2984261 20230624

[44] Eberle, R. J. et al. Riboflavin, a Potent Neuroprotective Vitamin: Focus on Flavivirus and Alphavirus Proteases. *Microorganisms* 10, (2022). 10.3390/microorganisms10071331 10.3390/microorganisms10071331 PMC9315535 35889050

