# Supplementary material for: In silico screening and experimental validation identify riboflavin as an RNA-targeted antiviral against SARS-CoV-2
Source: Sci Rep. 2025 Aug 22;15:30935. doi: 10.1038/s41598-025-16949-8 (PMC12373729; doi:10.1038/s41598-025-16949-8)
Supplement: Supplementary file 1 — Supplementary Material 1 [file 41598_2025_16949_MOESM1_ESM.docx]

**Supplementary Table S1.** **Chemicals predicted to bind to RNA secondary structure motifs according to the RNALigands database^1^**

| Name | Sequence, Secondary structure | Binding sites | Motif | PDB (score) | Chemical |
| --- | --- | --- | --- | --- | --- |
| SARS-CoV-2-Conserved-Structured-8 | UUUCGAUCUCUUGUAGAUCUGUUCUCUAAACGAACUUUAAAAUCUGUGUGGCUGUCACUCGGCUGCAUGCUUAGUGCACUCACGCAGUAUAAUUAAUAACUAAUUACUGUCGUUGACA  ....(((((.....))))).........(((((..........(((((((..((.((((.(((.....))).))))))..)))))))..((((((.....))))))...))))).... | 5′UTR | I: (G,U) C (U,A) C, B: (G,C) U (C,G), H: (C,G) UAAUUACU, M: (C,G) U (G,C) UUUAAAAU (C,G) UAUAA! (U,A) | G4P(24), GLY(86), SAH(21), G4P(57) |  |
| SARS-CoV-2-Conserved-Structured-34 | AAUCUGUGUGGCUGUCACUCGGCUGCAUGCUUAGUGCACUCACGCAGUAUAAUUAAUAACUAAUUACUGUCGUUGACAGGACACGAGUAACUCGUCUAUCUUCUGCAGGCUGCUUACG  ...(((((((..((.((((.(((.....))).))))))..)))))))..((((((.....))))))..((.(((..(((((.(((((...))))).....)))))..))).))..... | 5′UTR | I: (G,U) C (U,A) C, B: (G,C) U (C,G), I: (U,G) C (G,C) U, I: (G,C) UU (G,C) UU, B: (G,C) GAC (A,U), H: (G,C) UAA, M: (G,C) UA (U,A) (C,G) | G4P(24), GLY(86), **TOA(20),** 2BA(32), L94(67), **4PQ(23),** **2BA(59)** |  |
| SARS-CoV-2-Conserved-Structured-36 | CACGCAGUAUAAUUAAUAACUAAUUACUGUCGUUGACAGGACACGAGUAACUCGUCUAUCUUCUGCAGGCUGCUUACGGUUUCGUCCGUGUUGCAGCCGAUCAUCAGCACAUCUAGGU  ...((((((..((((.....)))))))))).((((((((((.(((((...))))).....)))))..((((((.(((((......)))))..)))))).....))))).......... | 5′UTR | B: (G,C) U (C,G), H: (G,C) UAA, I: (U,A) CUAUC (U,A) C, I: (C,G) U (U,G) UU, H: (G,C) UUUCGU | GLY(86), **TOA(92),** 4PQ(23), **SAH(39**), SAM(32), **HPA(99)** | Hypoxanthine  3-ammonio-3-deoxy-alpha-D-glucopyranose |
| SARS-CoV-2-Conserved-Structured-16 | GGACACGAGUAACUCGUCUAUCUUCUGCAGGCUGCUUACGGUUUCGUCCGUGUUGCAGCCGAUCAUCAGCACAUCUAGGUUUCGUCCGGGUGUGACCGAAAGGUAAGAUGGAGAGCCUUG  .....((((...(((.((((((((.....((((((.(((((......)))))..)))))).........(((((((.((......)))))))))(((....)))))))))))))).)))) | 5′UTR | I: (G,C) UAA (C,G) C, B: (C,G) G (U,A), I: (C,G) U (U,G) UU, H: (G,C) UUUCGU, B: (U,G) A (G,C), H: (G,C) UUUCGU, H: (C,G) GAAA, M: (U,A) CUGCA (G,C) GAUCAUCAG (C,G) (A,U) | G4P(45), TOA(54), SAM(32), HPA(99), NMY(79), **HPA(99),** AMZ(86), G4P(14) | Hypoxanthine  Neomycin |
| SARS-CoV-2-Conserved-Structured-1 | UGUAAGCACAAGCUGAUGAGUACGAACUUAUGUACUCAUUCGUUUCGGAAGAGACAGGUACGUUAAUAGUUAAUAGCGUACUUCUUUUUCUUGCUUUCGUGGUAUUCUUGCUAGUUACAC  ((((((((.((((.((((((((((......)))))))))).)))).((((((((..(((((((((........))))))))))))))))).))).....(((((....))))).))))). | ORF1ab | I: (G,C) U (A,U) U, I: (C,G) U (G,U) C, B: (A,U) CA (G,U), I: (U,A) U (C,G) U, M: (A,U) C (A,U) C (G,C) U, M: (C,G) G (U,A) | GE3(93), FFO(24), OHX(15), GE3(88), **2BA(23),** **SAH(110)** | **S-Adenosyl-L-homocysteine** |
| SARS-CoV-2-Conserved-Structured-2 | UUACCAGAUCCAUCAAAACCAAGCAAGAGGUCAUUUAUUGAAGAUCUACUUUUCAACAAAGUGACACUUGCAGAUGCUGGCUUCAUCAAACAAUAUGGUGAUUGCCUUGGUGAUAUUGCU  (((((((...............(((((..(((((((.(((((((.....)))))))..))))))).))))).......(((.((((((.......))))))..))))))))))....... | ORF1ab | I: (G,C) AG (G,C) A, I: (U,A) A (U,A) CA | **2BA(123)**, GES(54) | **c-di-AMP sodium salt** |
| SARS-CoV-2-Conserved-Structured-3 | AAAAAGGCUGGUGGCACUACUGAAAUGCUAGCGAAAGCUUUGAGAAAAGUGCCAACAGACAAUUAUAUAACCACUUACCCGGGUCAGGGUUUAAAUGGUUACACUGUAGAGGAGGCAAAG  .....(.(((.(((((((.((.((.....(((....))))).))...))))))).))).)..(((((((((((...((((......)))).....))))))...)))))........... | ORF1ab | I: (G,C) G (U,A) A, I: (U,A) A (C,G) AAA, I: (U,A) G (A,U) G, B: (A,U) U (G,C), H: (U,A) AGCGAA, M: (G,C) ACAAUUAUA (U,A) | TPP(38), G4P(107), BFT(18), OHX(80), AMZ(13), **4BW(114)** | **cGAMP sodium salt** |
| SARS-CoV-2-Conserved-Structured-4 | GAUGUUGACACAGACUUUGUGAAUGAGUUUUACGCAUAUUUGCGUAAACAUUUCUCAAUGAUGAUACUCUCUGACGAUGCUGUUGUGUGUUUCAAUAGCACUUAUGCAUCUCAAGGUCUA  (((.((((..((((..(..(.(.((((.((((((((....)))))))).....)))).).)..).....))))..(((((...((.(((((.....))))).)).))))))))).))).. | ORF1ab | I: (G,C) U (G,C) U, I: (C,G) UC (U,A) AU, B: (A,U) C (G,C), B: (U,A) GCA (U,A), M: (A,U) CAUU (U,A) U (G,C) (A,U) | GE3(102), G4P(64), LYS(86), L94(20), **ADE(136)** | **Adenine** |
| SARS-CoV-2-Conserved-Structured-5 | AACUAUAGGUCCAGACAUGUUCCUCGGAACUUGUCGGCGUUGUCCUGCUGAAAUUGUUGACACUGUGAGUGCUUUGGUUUAUGAUAAUAAGCUUAAAGCACAUAAAGACAAAUCAGCUCA  ......(((.((.((((.((((....)))).))))))......)))(((((..(((((.......((.((((((((((((((....)))))).)))))))).))..))))).)))))... | ORF1ab | B: (C,G) A (G,C), I: (A,U) U (G,C) U, H: (U,A) GAUA, B: (C,G) U (U,G), I: (C,G) A (U,G) A, I: (A,U) A (U,A) AA | **NMY(128)**, GE3(65), AMZ(37), GLY(70), HPA(13), GE3(87) | **Neomycin solution** |
| SARS-CoV-2-Conserved-Structured-6 | CACUUAACGAUCUAAAUGAAACUCUUGUUACAAUGCCACUUGGCUAUGUAACACAUGGCUUAAAUUUGGAAGAAGCUGCUCGGUAUAUGAGAUCUCUCAAAGUGCCAGCUACAGUUUCUG  ..........(((((((..((..(.(((((((..(((....)))..)))))))...)..))..)))))))((((((((((.(((((.((((....))))..))))))))...))))))). | ORF1ab | I: (U,A) GA (A,U) AA, I: (A,U) C (U,G) C, I: (C,G) U (U,A) CAU, I: (A,U) AU (G,C) UA, H: (C,G) ACUU, B: (U,A) C (G,C), H: (G,C) AUCU, I: (A,U) AA (G,U) A, B: (C,G) UAC (A,U) | 2BA(39), G4P(54), G4P(64), 2BA(99), MGX(11), **4PQ(115),** AMP(24), GE3(64), C2E(38) | **5-Hydroxy-L-tryptophan** |
| SARS-CoV-2-Conserved-Structured-7 | CUGAUAGAGACCUUUAUGACAAGUUGCAAUUUACAAGUCUUGAAAUUCCACGUAGGAAUGUGGCAACUUUACAAGCUGAAAAUGUAACAGGACUCUUUAAAGAUUGUAGUAAGGUAAUCA  ..(((....(((((....((((((((((.(((...((.(((((((((((((((....)))))).)).))).)))))).))).)))))).....(((....)))))))...))))).))). | ORF1ab | I: (G,C) A (C,G) A, I: (A,U) A (U,A) A, B: (U,A) A (C,G), B: (A,U) A (G,C), B: (G,C) U (C,G), H: (U,A) AGGA, B: (G,C) C (A,U), B: (A,U) C (U,A), B: (U,A) A (C,G), M: (U,A) UUAAA (G,C) | GE3(71), HPA(90), FH8(120), **0EC(117),** GLY(86), EKJ(56), LYS(86), SAM(53), FH8(120) | **6,7-DIMETHOXY-2-(1-PIPERAZINYL)-4-QUINAZOLINAMINE** |
| SARS-CoV-2-Conserved-Structured-8 | GGAAGUCACACCUUCGGGAACGUGGUUGACCUACACAGGUGCCAUCAAAUUGGAUGACAAAGAUCCAAAUUUCAAAGAUCAAGUCAUUUUGCUGAAUAAGCAUAUUGACGCAUACAAAAC  ....((((.(((..((....)).)))))))........((((.......((((((.......))))))..............((((...((((.....))))...))))))))....... | ORF1ab | H: (G,C) UCACAC, B: (U,G) UG (A,U), M: (C,G) GGGAAC (G,U) CAAA (U,A) AUUUCAAAGAUCAA! (G,C) | HPA(12), SAM(30), **B1Z(117)** | **Coenzyme B12** |
| SARS-CoV-2-Conserved-Structured-9 | GGUUUAACUUCUAUUAAAUGGGCAGAUAACAACUGUUAUCUUGCCACUGCAUUGUUAACACUCCAACAAAUAGAGUUGAAGUUUAAUCCACCUGCUCUACAAGAUGCUUAUUACAGAGCA  ((...((((((.(((.....((((((((((....)))))).)))).(((..(((((........))))).)))))).))))))....))....(((((..((........))..))))). | ORF1ab | I: (U,A) U (A,U) U, H: (C,G) AACU, B: (C,G) U (U,A), I: (U,A) U (A,U) U, I: (U,A) AC (A,U) AC, M: (C,G) UAUUAAAUG (G,C) (A,U) U, M: (C,G) CACC (U,A) | GE3(46), AMP(62), GLY(85), GE3(46), SAH(71), **I2A(121),** GNG(24) | **Hydroxocobalamin** |

Abbreviations: **G4P**, GUANOSINE-5′,3′-TETRAPHOSPHATE; **2BA**, (2R,3R,3aS,5R,7aR,9R,10R,10aS,12R,14aR)-2,9-bis(6-amino-9H-purin-9-yl)octahydro-2H,7H-difuro[3,2-d:3′,2′-j][1,3,7,9,2,8]tetraoxadiphosphacyclododecine-3,5,10,12-tetrol 5,12-dioxide; **L94,** N′-{(Z)-amino[4-(amino{[3-(dimethylammonio)propyl]iminio}methyl)phenyl]methylidene}-N,N-dimethylpropane-1,3-diaminium; **NMY**, NEOMYCIN; **FMN**, FLAVIN MONONUCLEOTIDE; **B1Z**, adenosylcobalamin; **GLY**, GLYCINE; **TOA**, 3-ammonio-3-deoxy-alpha-D-glucopyranose; **4PQ**, 5-hydroxy-L-tryptophan; **SAH**, S-ADENOSYL HOMOCYSTEINE; **SAM**, S-ADENOSYL METHIONINE; **HPA**, HYPOXANTHINE; **C2E**, 9,9′-[(2R,3R,3aS,5S,7aR,9R,10R,10aS,12S,14aR)-3,5,10,12-tetrahydroxy-5,12-dioxidooctahydro-2H,7H-difuro[3,2-d:3′,2′j][1,3,7,9,2,8]tetraoxadiphosphacyclododecine-2,9-diyl]bis(2-amino-1,9-dihydro-6H-purin-6-one); **ARG**, ARGININE; **LYS**, LYSINE; **GES**:3-deoxy-4-C-methyl-3-(methylamino)-beta-L-arabinopyranose; **AMZ**, AMINOIMIDAZOLE 4-CARBOXAMIDE RIBONUCLEOTIDE. The compounds highlighted in bold represent the candidates selected based on our screening criteria.

**Supplementary Table S2. CC_50_ and IC_50_ of test compounds in Vero E6 cells infected with SARS-CoV-2**

| **Test compound** | **CC_50_ (μM)** | **IC_50_ (μM)** |
| --- | --- | --- |
| c-di-AMP sodium salt (2BA) | >100 | - |
| 5-Hydroxy-L-tryptophan (4PQ) | >100 | - |
| Hypoxanthine (HPA) | >100 | - |
| cGAMP sodium salt | >100 | - |
| Adenine (ADE) | >100 | - |
| 6,7-dimethoxy-2-(1-piperazinty)-4-quinazolinamine (OEC) | >100 | - |
| Coenzyme B12 (B1Z) | >100 | - |
| **Riboflavin 5′-monophosphate sodium salt hydrate (FMN)** | **98.86** | **59.409** |
| S-Adenosyl-L-homocysteine (SAH) | >100 | - |
| Neomycin solution (NMY) | >100 | - |
| Hydroxocobalamin (HPA) | >100 | - |
| **Remdesivir** | **>100** | - |

**Supplementary Table S3. CC_50_ and IC_50_ of remdesivir and FMN administered at different points in Vero E6 cells infected with SARS-CoV-2**

| **Test compound** | **CC_50_ (μM)** | **IC_50_ (μM)** | | |
| --- | --- | --- | --- | --- |
|  |  | **Pre-infection** | **During infection** | **Post-infection** |
| Riboflavin 5′-monophosphate sodium salt hydrate (FMN) | >100 | - | 59.41 | - |
| Remdesivir | 98.86 | <6.111 | 25.81 | 3.471 |

Vero E6 cells were treated with indicated doses (0–100 μM) of the test compounds, remdesivir or FMN. Their CC_50_ and IC_50_ values are summarized in this table. The test compound was added 2 h before, simultaneously with, or 2 h after SARS-CoV-2 inoculation. In the first case, cells were first incubated with the compound at 37°C for 2 h, washed three times with PBS, and infected with SARS-CoV-2 for 2 h. In the second case, cells were simultaneously incubated with SARS-CoV-2 and the test compound for 2 h at 37°C. The mixture was then removed, and the cells were washed three times with PBS before fresh medium was added. In the last case, the cells were infected with SARS-CoV-2 for 2 h, washed three times with PBS, and incubated in a medium containing the test compound. Viral titers were determined at 48 h post-infection. Cell viability and antiviral activity were evaluated using the EZ-Cytox kit and a plaque assay with crystal violet staining, respectively.

**Supplementary Figure S1. Evaluation of the antiviral efficacy of FMN, a riboflavin derivative.** Vero E6 cells were treated with indicated doses of remdesivir or FMN. The half-maximal inhibitory concentration (IC_50_) values of these compounds are indicated on the curves. Cells were maintained in drug-containing media for 48 h. Evaluation of antiviral activity was performed using a plaque assay with crystal violet staining.


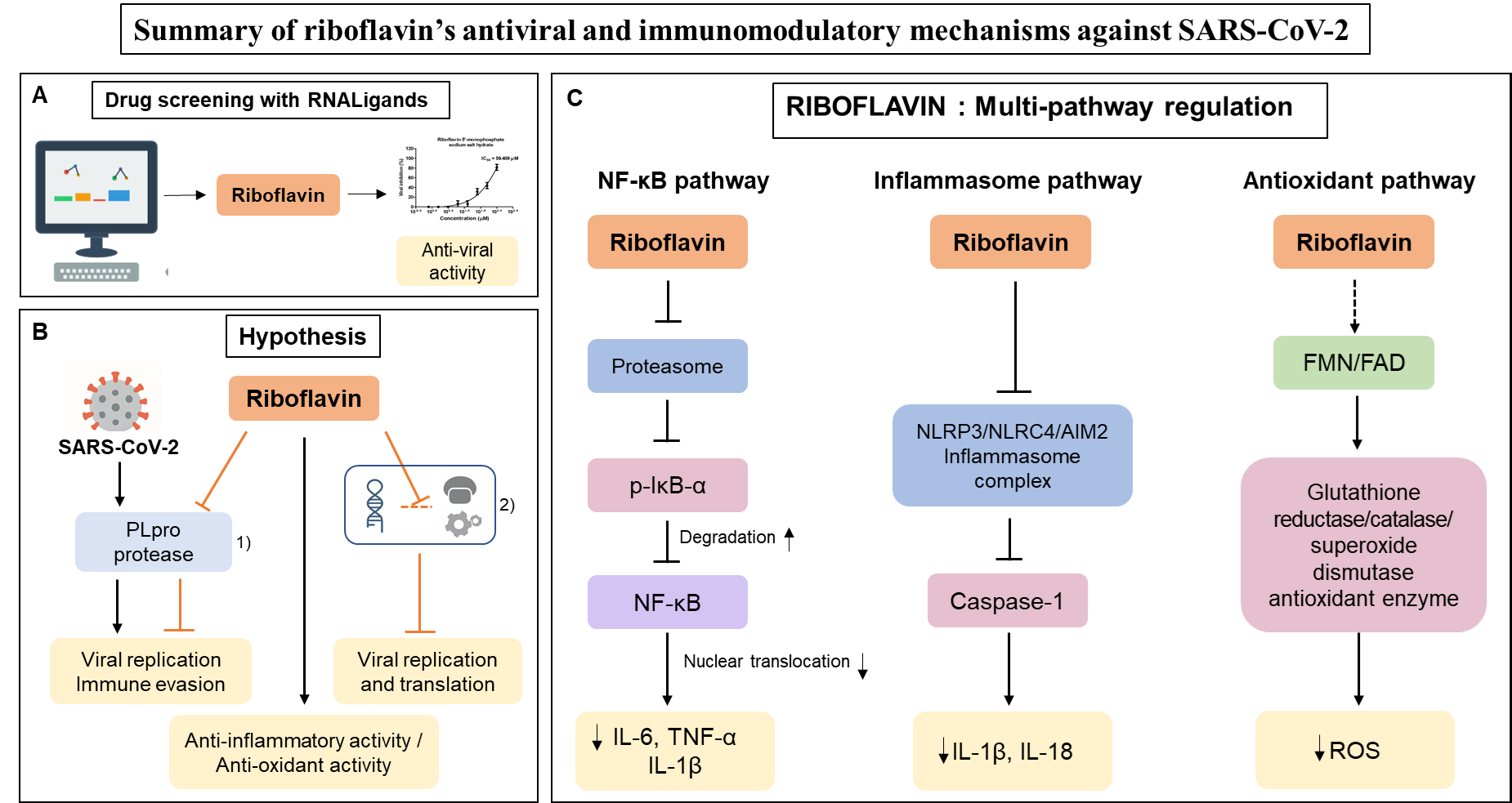


**Supplementary Figure S2. Schematic summary of riboflavin’s antiviral and immunomodulatory mechanisms against SARS-CoV-2.** (A) Schematic representation of the drug discovery workflow used in this study. The pipeline begins with in silico screening to identify potential RNA-binding candidates using the RNALigands program, followed by in vitro validation of antiviral activity against SARS-CoV-2. (B) Proposed antiviral mechanisms of riboflavin against SARS-CoV-2. This panel illustrates two modes of action: 1) inhibition of the viral papain-like protease (PLpro) at high concentrations (>50 µM), as reported in previously^2^; 2) the main hypothesis of this study proposes that riboflavin in directly binds to a structural motif within the 5' untranslated region (5' UTR) of the viral RNA, which may inhibit viral translation and/or replication by disrupting RNA-ribosome or replication complex binding. (C) Schematic overview of reported immunomodulatory mechanisms of riboflavin, as reported in referenced literature^3-7^. These schematic highlights riboflavin's role in modulating key inflammatory pathways, providing a broader context for its therapeutic potential beyond direct antiviral activity.

**Reference**

1. Rangan, R. *et al.* RNA genome conservation and secondary structure in SARS-CoV-2 and SARS-related viruses: a first look. *Rna* **26**, 937–959 (2020).

2. Akasov, R. A. *et al.* Evaluation of molecular mechanisms of riboflavin anti-COVID-19 action reveals anti-inflammatory efficacy rather than antiviral activity. *Biochim Biophys Acta Gen Subj* **1868**, 130582, doi:10.1016/j.bbagen.2024.130582 (2024).

3. Ahn, H. & Lee, G. S. Riboflavin, vitamin B2, attenuates NLRP3, NLRC4, AIM2, and non-canonical inflammasomes by the inhibition of caspase-1 activity. *Sci Rep* **10**, 19091, doi:10.1038/s41598-020-76251-7 (2020).

4. Suwannasom, N., Kao, I., Pruss, A., Georgieva, R. & Baumler, H. Riboflavin: The Health Benefits of a Forgotten Natural Vitamin. *Int J Mol Sci* **21**, doi:10.3390/ijms21030950 (2020).

5. Dricot, C. *et al.* Riboflavin for women's health and emerging microbiome strategies. *NPJ Biofilms Microbiomes* **10**, 107, doi:10.1038/s41522-024-00579-5 (2024).

6. Tay, M. Z., Poh, C. M., Renia, L., MacAry, P. A. & Ng, L. F. P. The trinity of COVID-19: immunity, inflammation and intervention. *Nat Rev Immunol* **20**, 363–374, doi:10.1038/s41577-020-0311-8 (2020).

7. Hu, B., Huang, S. & Yin, L. The cytokine storm and COVID-19. *J Med Virol* **93**, 250–256, doi:10.1002/jmv.26232 (2021).
